# Serum syndecan-1 concentration in hemolysis, elevated liver enzymes, and low platelets syndrome: A case report

**DOI:** 10.3389/fmed.2023.1111139

**Published:** 2023-03-14

**Authors:** Ayane Nishio, Ryo Kamidani, Hideshi Okada, Keiko Suzuki, Kodai Suzuki, Takahito Miyake, Haruka Okamoto, Tomoaki Doi, Akio Suzuki, Shozo Yoshida, Shinji Ogura

**Affiliations:** ^1^Advanced Critical Care Center, Gifu University Hospital, Gifu, Japan; ^2^Abuse Prevention Center, Gifu University Graduate School of Medicine, Gifu, Japan; ^3^Department of Infection Control, Gifu University Graduate School of Medicine, Gifu, Japan; ^4^Department of Pharmacy, Gifu University Hospital, Gifu, Japan

**Keywords:** HELLP syndrome, syndecan-1, vascular endothelial injury, glycocalyx, case report

## Abstract

**Background:**

Hemolysis, elevated liver enzymes, and low platelets (HELLP) syndrome occurs in pregnant and postpartum individuals. We observed serum syndecan-1 (SDC-1) levels, which is a component of the glycocalyx, in a patient with HELLP syndrome from admission to the postpartum period and examined their association as reflecting the pathophysiology related to endothelial injury.

**Case presentation:**

A 31-year-old primiparous female patient without a previous medical history at a gestational age of 37 weeks and 6 days was transferred to our hospital the morning after a visit to a previous hospital with headache and nausea. Elevated transaminase, platelet count, and proteinuria were noted. Head magnetic resonance imaging revealed a caudate nucleus hemorrhage and posterior reversible encephalopathy syndrome. After she delivered her newborn through an emergency cesarean section, she was admitted to the intensive care unit. On day 4 post-delivery, the patient’s D-dimer concentration was elevated, and contrast-enhanced computed tomography was performed. The results indicated pulmonary embolism, and heparin administration was initiated. The serum SDC-1 level was highest on day 1 post-delivery and quickly decreased subsequently; however, it remained elevated during the postpartum period. Her condition gradually improved, and she was extubated on day 6 and discharged from the ICU on day 7 post-delivery.

**Conclusion:**

We measured SDC-1 concentration in a patient with HELLP syndrome and found that the clinical course correlated with SDC-1 levels, indicating that SDC-1 is elevated immediately before and after pregnancy termination in patients with HELLP syndrome. Therefore, SDC-1 fluctuations, combined with the elevation of the D-dimer level, may be a potential marker for the early detection of HELLP syndrome and estimation of the syndrome’s severity in the future.

## 1. Introduction

Hemolysis, elevated liver enzymes, and low platelets (HELLP) syndrome encompass a group of clinical manifestations that occur in pregnant and postpartum individuals, which progresses to multiple organ dysfunction and results in severe clinical outcomes, such as stillbirth and death. Furthermore, it has been reported that vascular endothelial disorder is involved in HELLP syndrome (1), with the endothelial glycocalyx, which is composed of glycoproteins and polysaccharides and covers the healthy vascular endothelium, also facing potential impairment.

Here, we report the case of a patient with HELLP syndrome. Serum syndecan (SDC)-1, which is a component of the glycocalyx and a well-known marker of vascular endothelial glycocalyx (2, 3), was measured from admission to the postpartum period. We examined the association of SDC-1 levels with the symptoms of HELLP syndrome as a reflection of the disease state with endothelial injury.

## 2. Case description

A 31-year-old primiparous female patient of 37 weeks and 6 days gestational age presented to a previous hospital with headaches and nausea the night before admission. She had no history of eclampsia, hypertension, diabetes, systemic lupus erythematosus, or anti-phospholipid antibody syndrome, and there was no family medical history. Furthermore, no abnormalities were observed on her head computed tomography (CT) scan, and consequently, she left the hospital. However, the symptoms persisted and she experienced convulsions on the morning of re-admission to the previous hospital.

## 3. Case timeline

**Figure 1:**
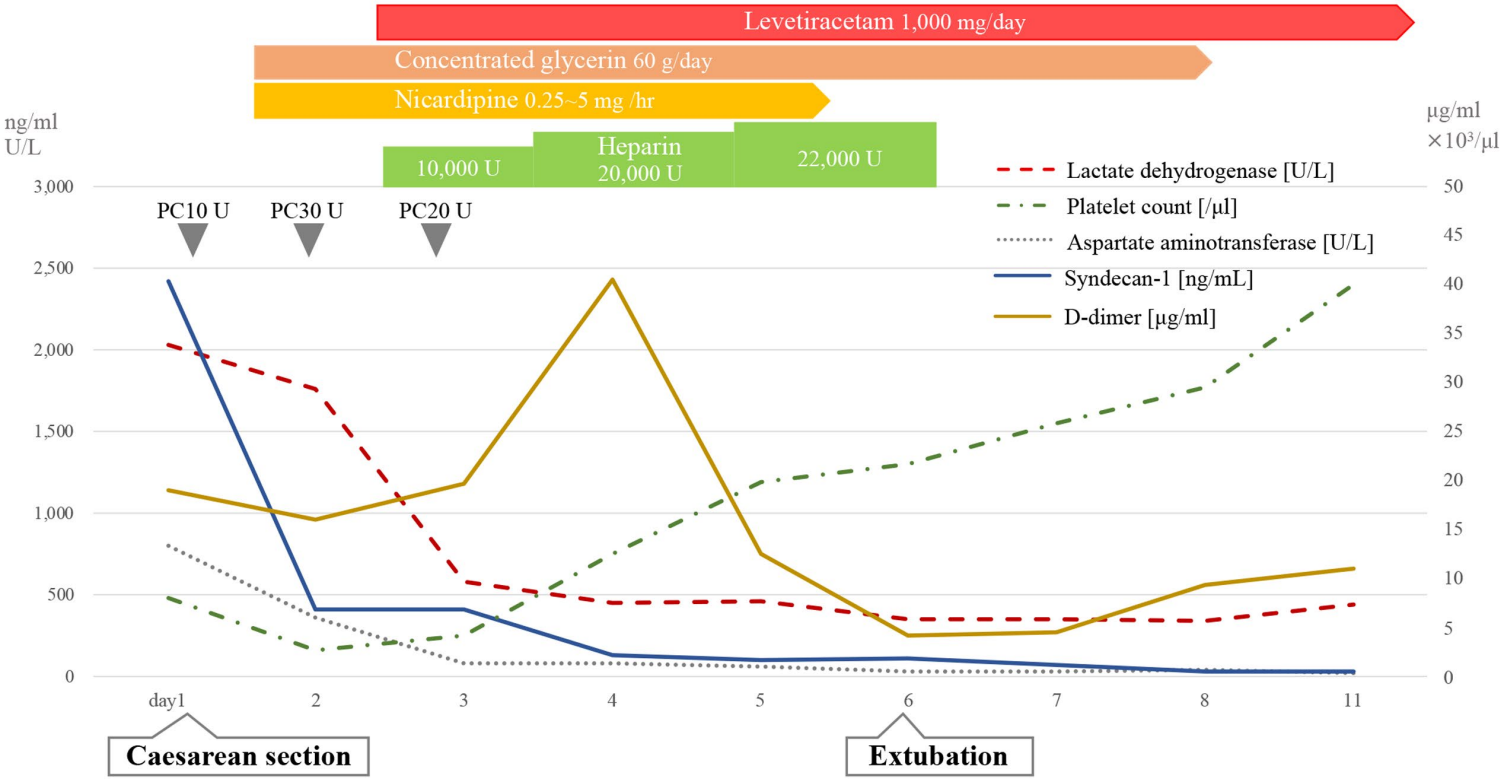
Clinical course. PC, platelet concentrate; LD, lactic acid dehydrogenase; PLT, platelet counts; AST, aspartate aminotransferase; SDC-1, syndecan-1.

Clinical course shown in Figure 1.

## 4. Diagnostic assessment

The patient was transferred to our hospital based on her blood test results, in which HELLP syndrome was suspected. She developed convulsions on arrival and was administered diazepam for treatment. Physical examination revealed a respiratory rate of 25 breaths per minute, heart rate of 95 beats per minute, blood pressure of 169/111 mmHg, and body temperature of 36.5°C. No new skin findings were observed. Blood test results were as follows: aspartate aminotransferase, 799 U/L (normal range 10–40 U/L); alanine aminotransferase, 326 U/L (normal range 5–40 U/L); lactate dehydrogenase, 2,031 U/L (normal range 124–222 U/L); and platelet count, 8,000/μL (normal range 131,000–362,000/μL) (Table 1). The serum SDC-1 level, which was measured using an enzyme-linked immunosorbent assay (950.640.192; Diaclone, Besancon, Cedex, France), was 2,418 ng/mL. Proteinuria was also observed, and the plain abdominal CT scan showed no intestinal wall thickening or increased mesenteric fatty tissue density.

**Table 1:** Laboratory findings on admission.

< Biochemistry >			< Complete Blood Count >		
Albumin	2.6	g/dL	White Blood Cells	23,150	/uL
Creatinine Kinase	378	IU/L	Red Blood Cells	3.38 × 10^6^	/uL
AST	799	IU/L	Hemoglobin	11.5	g/dL
ALT	326	IU/L	Hematocrit	32.2	%
LDH	2,031	IU/L	Platelet	80 × 10^3^	/uL
ALP	472	IU/L			
γ-GTP	21	IU/L	**< Coagulation Status >**		
Triglyceride	152	mg/dL	APTT	33.8	sec
Cholesterol	185	mg/dL	PT-INR	1.21	
Uric acid	9.8	mg/dL	Fibrinogen	169	mg/dL
Total bilirubin	2.2	mg/dL	FDP	35.9	μg/dL
Creatinine	1.27	mg/dL	D-dimer	19.1	μg/dL
BUN	21.9	mg/dL			
Sodium	136	mg/dL	**< Urinalysis >**		
Potassium	5.3	mEq/L	Protein (qualitative)	3+	
Chloride	108	mEq/L	Protein (qualitative)	300	mg/dL
Magnesium	4.3	mg/dL			
Calcium	8.3	mg/dL			
Glucose	237	mg/dL			
CRP	3.5	mg/dL			

AST, aspartate aminotransferase; ALT, alanine aminotransferase; LDH, lactic acid dehydrogenase; ALP, alkaline phosphatase; Γ-GTP, Γ-glutamyl transpeptidase; BUN, blood urea nitrogen; HbA1c, hemoglobin A1c; CRP, C-reactive protein; APTT, activated partial thromboplastin time; PT-INR, prothrombin time-international normalized ratio; FDP, fibrin degradation product; FiO_2_, fraction of inspiratory oxygen. Values in red or blue fonts indicate that the values exceed or fall below the normal limits, respectively.

**Figure 2:**
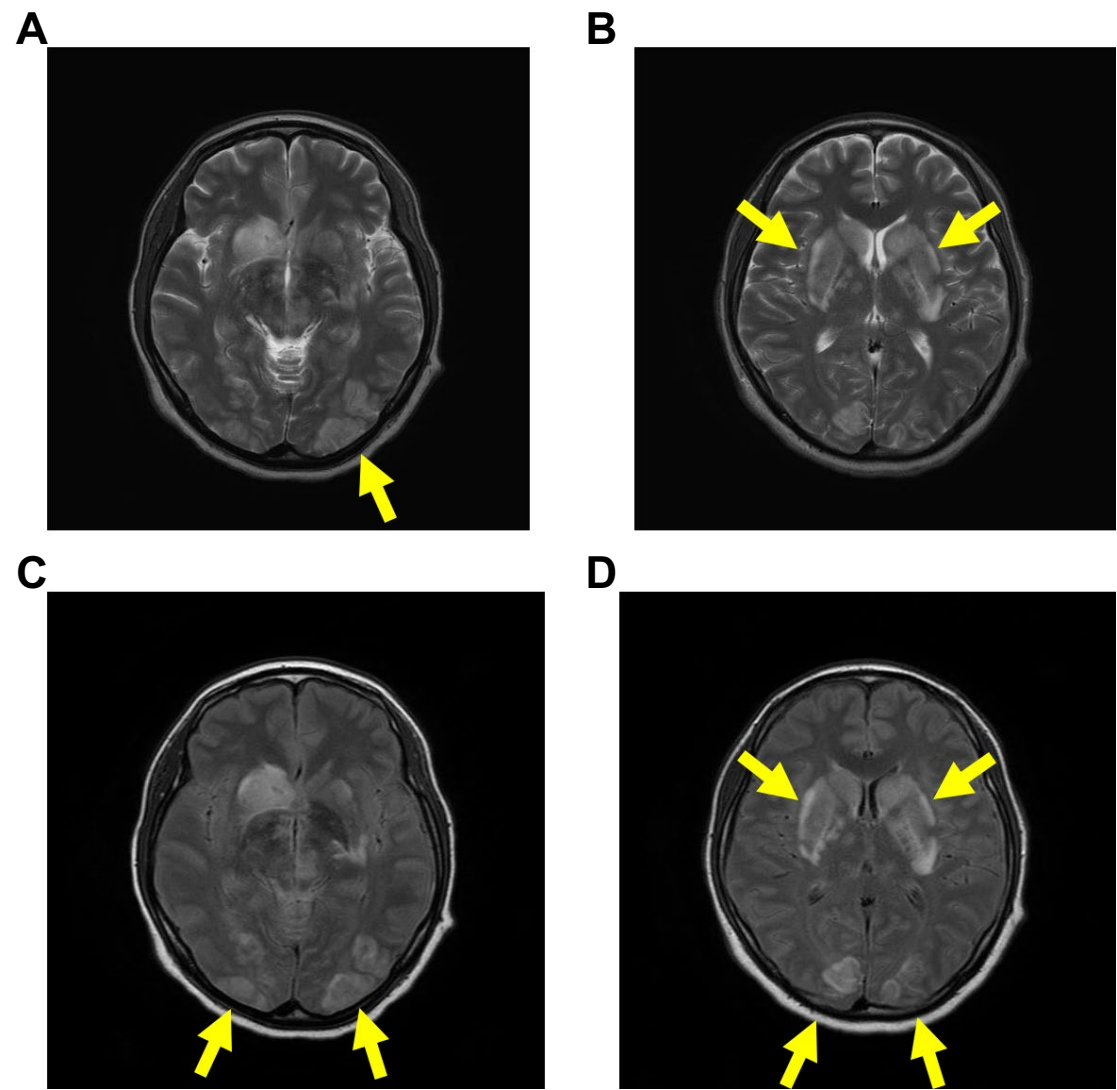
Head MRI on admission. The head MRI reveals caudate nucleus hemorrhage and PRES, with hyperintense signals on T2 **(A,B)** and FLAIR **(C,D)** sequences in the bilateral occipital regions, caudate nucleus, and putamen. Abbreviations: MRI, magnetic resonance imaging; PRES, posterior reversible encephalopathy syndrome; FLAIR, fluid-attenuated inversion recovery.

The patient was immediately intubated in the emergency department and delivered a newborn through cesarean section (CS) in the operating room. Additionally, head magnetic resonance imaging (MRI) showed a caudate nucleus hemorrhage and posterior reversible encephalopathy syndrome with hyperintense signals on T2 and fluid-attenuated inversion recovery (FLAIR) sequences in the bilateral occipital regions, caudate nucleus, and putamen (Figure 2). In this case, autoimmune antibodies measuring, ADAMTS13 activity measuring, blood smear, and liver biopsy were not performed.

She remained sedated and was administered platelet products, antihypertensive agents, anticonvulsants, and glycerol in the intensive care unit (ICU).

On day 4 post-delivery, her D-dimer level increased during routine blood tests, and pulmonary thromboembolism (PE) was confirmed through contrast-enhanced CT. We therefore initiated treatment with continuous intravenous heparin (10,000–22,000 U/day). Serum SDC-1 levels were the highest on day 1 post-delivery and rapidly decreased; however, they remained high during the postpartum period. Her condition gradually improved, and she was extubated on day 6, discharged from the ICU on day 7, and discharged on day 17 after admission (Figure 1).

## 5. Discussion

In this case, we measured SDC-1 concentration to aid in predicting disease states in future cases of HELLP syndrome. SDC-1 levels decreased substantially after CS as observed in healthy pregnant patients, but remained relatively high after pregnancy termination. Combined with the elevated D-dimer level, which is also a marker of vascular endothelial injury, this suggests that SDC-1 may be an early predictive marker of vascular endothelial injury in HELLP syndrome. However, as this was difficult to determine in this study alone, further validation studies are required, such as measuring SDC-1 beginning from a healthy period during non-pregnancy and through a portion of the lactation period post-partum.

The sugar-protein glycocalyx completely coats the healthy vascular endothelial cells. Additionally, it plays a key role in microvascular and endothelial physiology by influencing microvascular tone regulation and endothelial permeability, as well as inhibiting intravascular thrombosis. The SDC family can also be found in the endothelial glycocalyx. It includes core proteins in heparan sulfate proteoglycan and comprises SDC-1 to SDC-4 according to their discovery and the tissue of origin.

SDC-1 is the most extensively studied integral membrane heparan sulfate proteoglycan. In clinical studies, serum SDC-1 has been used as an endothelial injury marker for several diseases, including sepsis, cardiovascular disease, and acute kidney injury (1, 4–7). A previous report indicated that the median serum SDC-1 concentration was 19.3 ng/ml in healthy participants (8). The glycocalyx is abundantly expressed within the placenta, and SDC-1 is confined to the syncytiotrophoblast apical membrane and syncytiotrophoblast cytoplasm, as revealed by immunohistochemistry (9). It has also been reported that SDC-1 concentrations correlate with chorioallantoic cell volume, increasing SDC-1 concentration with advancing gestation (10). Importantly, a previous study reported that SDC-1 concentration in pregnant women increases ˃100-fold and reaches its highest (median 1,415 ng/mL) at a gestational age of 37–41 weeks and drastically decreases at 7–11 h postpartum (11, 12).

HELLP syndrome is a hypertensive disorder that develops in the perinatal period and occurs in approximately 0.9% of pregnant women (13–15). Its pathophysiology is similar to that of preeclampsia, with vascular endothelial injury, increased inflammatory cytokines, fibrin deposition, and increased platelet activation and consumption (1, 16–23). Furthermore, it has been reported that von Willebrand factor antigen levels are elevated in patients with preeclampsia and HELLP syndrome, suggesting endothelial cell activation. HELLP syndrome has a number of differenial diagnoses. Since there was no medical history or pregnancy complications until she had eclampsia at 37 weeks and 6 days gestation, and no new skin findings, proteinuria, or colitis, we did not actively suspect systemic lupus erythematosus, antiphospholipid syndrome, or hemolytic uremic syndrome. However, ADAMTS13, blood smears, and liver biopsy at the time of CS should have been performed, but were not available in this case. Therefore, it is possible that thrombotic microangiopathy and acute fatty liver of pregnancy may have overlapped, and when HELLP syndrome is suspected, it is important to conduct differential diagnosis, including the above-mentioned tests, in parallel with treatment.

The disorder clinically manifests as hypertension, proteinuria, and consumptive thrombocytopenia, including the formation of platelet-rich thrombi (19). Typically, D-dimer levels have been used as a standard marker of vascular endothelial injury and coagulopathy for the early detection of HELLP syndrome (24, 25). However, D-dimer detection has low sensitivity and diagnostic accuracy as it can also be elevated in thromboembolism, gastrointestinal hemorrhage, cardiac arrest syndrome, trauma, and respiratory infections (25–27). Moreover, while D-dimer is also elevated in aging, obesity, hypercholesterolemia, and hypotriglyceridemia, this was not observed in this case study patient (28). Although immunoglobulin and autoantibodies were not measured, their influence on the D-dimer trend in this case was considered to be limited. In this case, PE was diagnosed through a contrast-enhanced CT scan on day 4. As such, we speculated that the D-dimer levels may have peaked when the thrombus formation began during prolonged vascular endothelial damage.

Maternal serum SDC-1 levels are lower in early-and late-onset preeclampsia than in healthy pregnant individuals throughout pregnancy, despite being strongly detected in placental SDC-1 immunostaining (9). After delivery, it generally decreases as in normal pregnancies; however, serum SDC-1 in preeclampsia remains higher than that reported in normal pregnancies (29). This has been hypothesized to be due to the decreased expression of syncytiotrophoblast SDC-1 in women who transition to preeclampsia or alterations of the cortical cytoskeletal actin network in the syncytiotrophoblast that reduce SDC-1 release into the blood. However, the mechanisms of pathogenesis remain largely unexplored (9, 11). Therefore, it is unclear whether the fluctuations in serum SDC-1 levels observed in this case are specific to HELLP syndrome or whether they are also observed in superimposed preeclampsia or preeclampsia with underlying severe endothelial damage. As such, we believe that it is currently difficult to make a strict distinction between these overlapping conditions.

In contrast, an observational study that compared endothelial glycocalyx shedding in healthy pregnant and non-pregnant women with HELLP syndrome showed significantly high prepartum serum SDC-1 concentrations in those with HELLP syndrome. Serum levels of other endothelial glycocalyx components, such as heparan sulfate, hyaluronic acid, and soluble tumor necrosis factor-a receptor (an inflammatory marker) were also elevated (12). Therefore, elevated levels of SDC-1 during the postpartum period in preeclampsia and HELLP syndrome may be caused by the endothelial damage associated with the disease progression. In this case, serum SDC-1 concentration was relatively high, although it decreased immediately after the termination of pregnancy, suggesting that a considerable amount of glycocalyx components may have been shed. Despite the patient receiving several platelet transfusions, although it is difficult to prove unequivocally that SDC-1 levels are unaffected by platelet concentrate transfusion, there is currently no clear evidence that they increased serum SDC-1 concentrations, and as such we believe that their effect on values is limited (30, 31). With regards to erythrocyte transfusions however, a previous study demonstrated that transfusion of a single sex-mismatched RBC unit was associated with higher SDC-1 levels than transfusion of a sex-matched RBC unit (32). Finally, as the patient required intensive care and various medications were administered, it is likely that drugs affected the patient, and future studies are needed to delineate the specific impact.

Upon reflection, the contrast-enhanced CT scan was performed due of the elevated D-dimer level, which lead to a pulmonary embolism diagnosis and subsequent anticoagulation therapy. After delivery however, the high SDC-1 levels suggested that anticoagulation therapy could be administered earlier, considering the prolonged systemic vascular endothelial cell damage on the mother’s side. As SDC-1 levels are also elevated in normal pregnancies, it will therefore be necessary in the future to establish cutoff values to distinguish between normal pregnancies and those associated with HELLP syndrome. Furthermore, will also be essential to investigate the use of SDC-1 levels for estimating the extent of its pathogenesis and severity.

## 6. Conclusion

We measured the SDC-1 concentration of a patient with HELLP syndrome to determine its predictive potential for identifying its disease states. In this case, SDC-1 levels were elevated, particularly after CS, reaffirming the finding that SDC-1 reflects vascular endothelial damage. Although D-dimer also reflects the occurrence of thrombosis, SDC-1 is less susceptible, suggesting that these two markers of vascular endothelial damage might be useful in daily practice to understand the severity and treatment reactivity of patients with HELLP syndrome. Unfortunately, we were unable to obtain samples prior to the onset of the disease, and it is therefore unclear whether SDC-1 accurately predicted the occurrence of HELLP syndrome in this case. However, as the pathophysiology is frequently associated with hypertension and systemic inflammation, SDC-1 may also be useful in predicting the occurrence of HELLP syndrome and can be a target for future studies.

## Data availability statement

The original contributions presented in the study are included in the article/supplementary material, further inquiries can be directed to the corresponding author.

## Ethics statement

Ethical review and approval were not required for this study in accordance with the local legislation and institutional requirements. Written informed consent to participate in this study was provided by the participants or their legal guardian/next of kin. Written informed consent was obtained from the individual(s), and minor(s)’ legal guardian/next of kin, for the publication of any potentially identifiable images or data included in this article.

## Author contributions

Treatment of the patient was performed by AN, RK, HiO, KoS, TM, HaO, TD, SY, and SO. Study concepts and designs were performed by AN, RK, and HiO. Data analysis was performed by AN, RK, HiO, KeS, KoS, TM, HaO, TD, AS, SY, and SO. The drafting and critical revision of the manuscript were performed by AN and RK. All authors contributed to the article and approved the submitted version.

## Conflict of interest

The authors declare that the research was conducted in the absence of any commercial or financial relationships that could be construed as a potential conflict of interest.

## Publisher’s note

All claims expressed in this article are solely those of the authors and do not necessarily represent those of their affiliated organizations, or those of the publisher, the editors and the reviewers. Any product that may be evaluated in this article, or claim that may be made by its manufacturer, is not guaranteed or endorsed by the publisher.
